# Omental cyst: a case report and review of the literature

**DOI:** 10.1186/s43159-021-00129-0

**Published:** 2021-12-02

**Authors:** Keenan J. Robbins, Ryan M. Antiel, Baddr A. Shakhsheer

**Affiliations:** 1 Section of Surgical Oncology, Department of Surgery, Washington University School of Medicine in St. Louis, Campus Box 8109, 660 S. Euclid Ave., St. Louis, MO 63100 USA; 2 Division of Pediatric Surgery, Department of Surgery, Washington University School of Medicine in St. Louis, Campus Box 8109, 4590 Children’s Place, Suite 9600, St. Louis, MO 63110 USA

**Keywords:** Cyst, Omental, Pediatric

## Abstract

**Background:**

Omental cysts are rare, predominantly occur in children, and often initially present with symptoms masquerading as other more common intra-abdominal pathologies. In this case report, we present the case of a child with an omental cyst that originated from the lesser sac. Due to the location of this cyst, resection presented unique technical challenges that have not been described in existing literature.

**Case presentation:**

A 4-year-old male patient presented with symptoms initially concerning for appendicitis. Ultrasound showed a normal appendix but a large volume of complex intraperitoneal fluid. Computed tomography subsequently demonstrated a large cystic structure spanning from the stomach to the bladder. The patient was taken to the operating room where a large omental cyst was found to originate from the lesser sac. The resection was difficult due to the thin wall of the cyst and the intimate association of the superior-most aspect of the cyst with the tail of the pancreas, but was ultimately successful.

**Conclusions:**

Omental cysts are rarely suspected before detection on abdominal imaging. Surgical resection is the treatment of choice, and complete resection can result in a recurrence-free postoperative course. Laparoscopic resection has been reported, but laparotomy is reasonable when a minimally invasive approach may not allow for a safe resection without rupture of the cyst. Anatomical characteristics of the cyst, as demonstrated in our case, can present challenges in the treatment of this otherwise benign entity.

## Background

Omental cysts are rare intra-abdominal masses that occur primarily in children. They often present with symptoms similar to other more common abdominal pathologies and are discovered incidentally on imaging. Herein, we discuss the case of a 4-year-old male who presented with abdominal pain and was discovered to have a large primary omental cyst originating from within the lesser sac. The location of this cyst presented unique technical challenges during surgical resection that have not been described in existing literature.

## Case presentation

A 4-year-old male with no significant past medical history presented to our emergency department with one day of worsening abdominal pain. The pain was diffuse but greatest in his periumbilical area. His mother also noticed new-onset abdominal distention. He demonstrated decreased appetite but had no emesis. His last bowel movement was the previous evening and was normal. Upon presentation, the patient was febrile to 38.7 °C. On exam, the patient’s abdomen was soft but distended with tenderness to palpation around the umbilicus and in the left hemiabdomen. Labs were significant for leukocytosis (WBC 20.1) but otherwise unremarkable. The patient was tested for influenza A/B and respiratory syncytial virus (RSV)—all of which were negative.

**Fig. 1:**
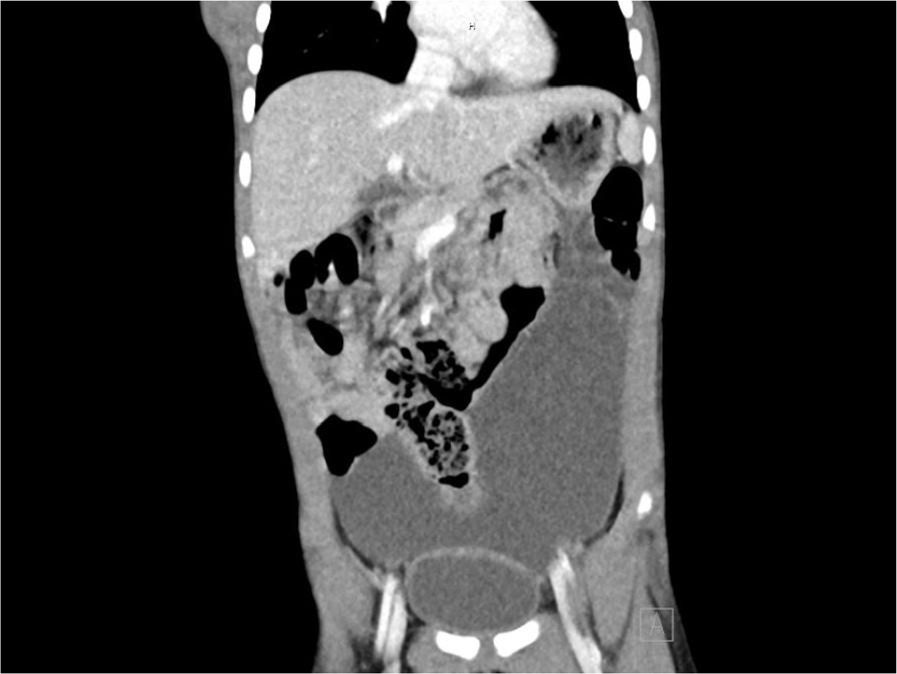
Computed tomography of the abdomen demonstrated a 7.3 × 11.7 × 14.9-cm cystic mass originating from the greater curve of the stomach and reaching the dome of the bladder

A transabdominal ultrasound was performed which demonstrated a normal appendix. Moderate volume complex ascites with particulate matter was also noted on this study. Given the unexplained presence of debris-containing intraperitoneal fluid, computed tomography (CT) of the abdomen and pelvis with intravenous contrast was obtained. This study showed a large cystic lesion in the left hemiabdomen, measuring 7.3 × 11.7 × 14.9 cm, which extended from the greater curvature of the stomach to the dome of the bladder (Fig. 1). The cyst was noted to exert mass effect on the small bowel and colon, but there was no evidence of obstruction.

**Fig. 2:**
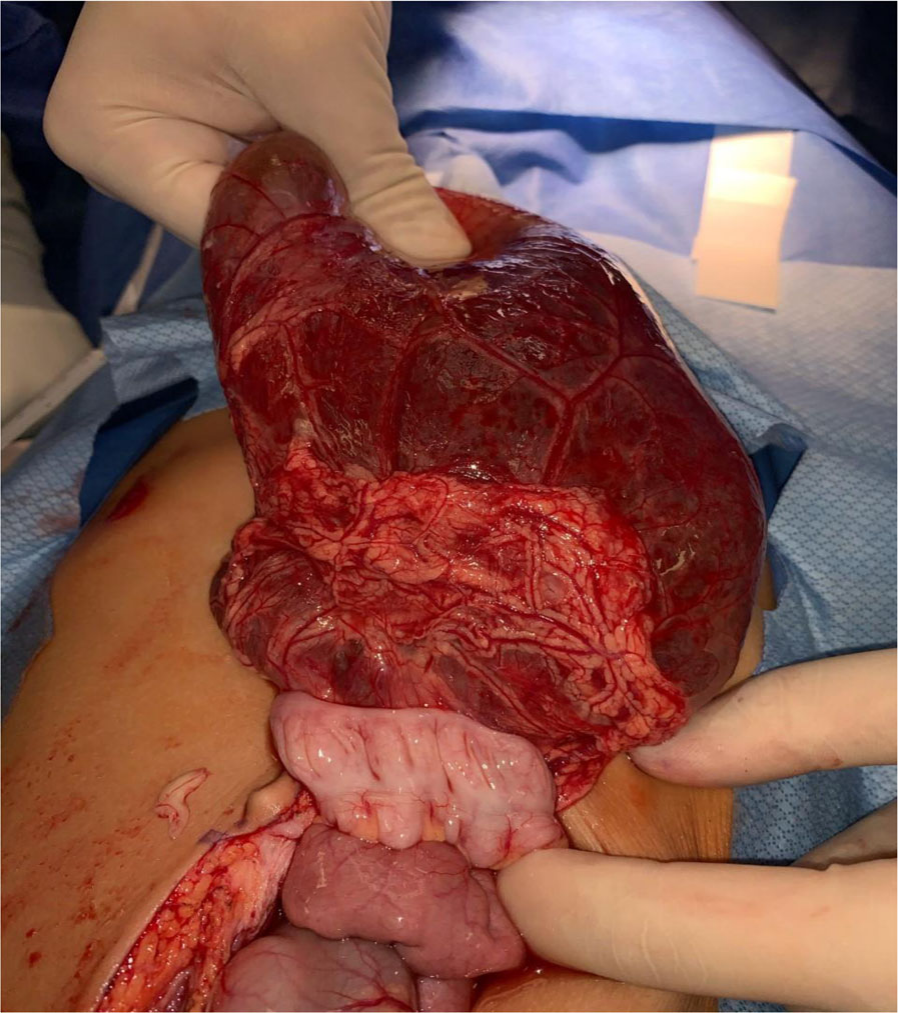
After being freed from the omentum laterally and inferiorly, the cyst was reflected cephalad. Note the adherence of the cyst to the transverse colon. The superior origin of the cyst was followed into the lesser sac, where it was intimately associated with the tail of the pancreas

**Fig. 3:**
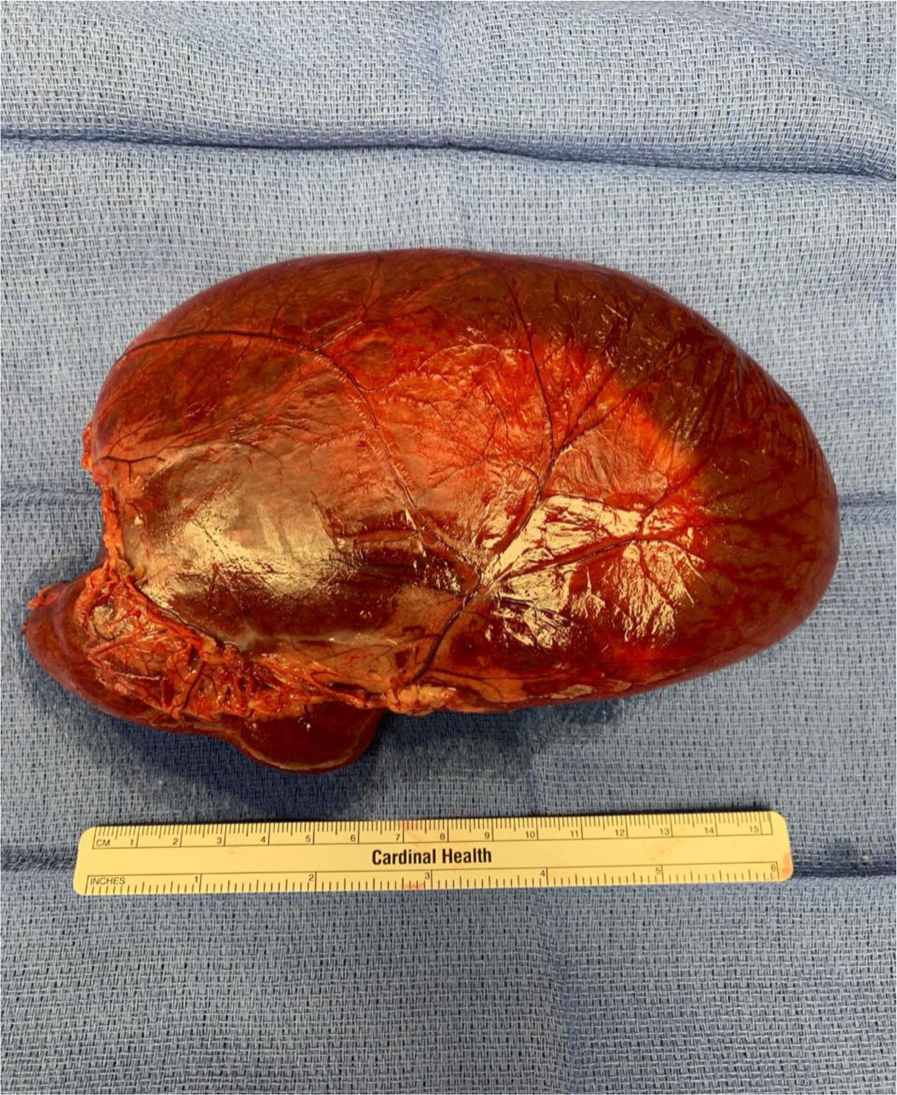
After careful separation from the tail of the pancreas, the cyst was removed intact and sent to pathology

**Fig. 4:**
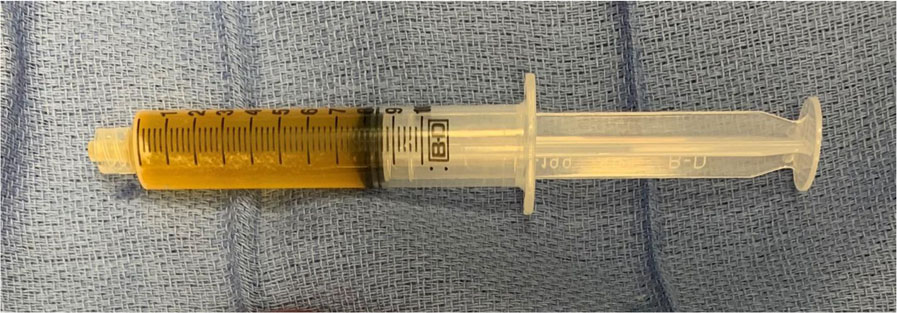
Serous fluid aspirated from cyst

The patient was admitted to the pediatric surgery service. The following day, the patient was taken to the operating room for exploratory laparotomy. Upon entering the peritoneal cavity a large thin-walled cyst was apparent, occupying the majority of the lower hemiabdomen. The cyst contained serous fluid and appeared to be multilocular. There were multiple solid components floating within the cystic fluid, all several centimeters in length. The cyst was carefully elevated out of the abdomen and traced to its origin in the lesser sac. The thin walls of the cyst necessitated careful handling to avoid rupture. The inferior and lateral borders of the cyst were continuous with the omentum, and the cyst was separated from the omentum using electrocautery and a LigaSure device (Medtronic, Minneapolis, Minnesota). The superior-most attachment of the cyst was then explored (Fig. 2). It appeared that the cyst was arising from the confluence of the transverse mesocolon and the inferior aspect of the tail of the pancreas. It was not clear if the cyst was in communication with the pancreatic ductal system, so the LigaSure device was jettisoned and meticulous sharp dissection was employed to free the cyst from the inferior aspect of the pancreatic tail. There was no bleeding or leakage of fluid from the pancreas, indicating that the ductal system was not involved. The cyst was then removed intact from the patient’s abdomen and placed on the back table (Fig. 3). Off the surgical field, fluid from the cyst was aspirated and sent for amylase, lipase, and triglycerides (Fig. 4). Results of these studies were not consistent with pancreatic or chylous fluid. Gross pathological analysis of the specimen found a 351-g cystic mass with dimensions of 15.0 × 11.0 × 4.0 centimeters. Congested vasculature and dilated lymphatic structures raised the question of whether this was originally of lymphangiomatous origin or whether these changes were due to torsion of the mass causing lymphatic and vascular congestion. There were solid components of necrotic fat suspended within the cystic fluid. Ultimately, this mass was felt to represent an omental cyst of indeterminate histologic origin. Immunohistochemical analysis was not performed.

Postoperatively, the patient returned to the surgical floor. His subsequent course was unremarkable and he was discharged on postoperative day number two. The patient was seen in clinic four weeks after discharge and was doing well. His mother felt that his appetite had improved compared to his preoperative state, perhaps indicating that the cyst was causing some degree of subclinical early satiety prior to presentation.

## Discussion

Omental and mesenteric cysts are rare entities, differentiated by the location in which they occur, with a reported incidence of one in twenty thousand admissions to a children’s hospital [1]. The majority of these cysts occur in children, with children under five years old accounting for 75% of cases in certain series [2]. Omental cysts are the rarer of the two, comprising between 14% and 21% of cysts of gastrointestinal origin, based on data from case series [3, 4]. A purported mechanism for the development of a primary omental cyst is the abnormal fusion of the omental bursa’s ventral and dorsal lamellae [5]. These cysts have been categorized based on their histologic characteristics. In a system proposed by de Perrot, omental/mesenteric cysts have been categorized into subgroups—lymphatic, mesothelial, enteric, urogenital—based on their histological origin [6]. Additional categories include mature cystic teratomas and pseudocysts. Lymphatic and mesothelial cysts are the most common and can be differentiated by the presence of loculations as well a propensity to arise from certain structures [7]. Presentation is often with abdominal pain or fullness and, in the search for more common pathologies, these cysts are usually diagnosed incidentally by abdominal imaging. A previous case report from Seattle Children’s Hospital notes that, while omental cysts are commonly mistaken for ascites on ultrasound, the presence of mass effect and the absence of intermixing between the fluid and loops of bowel may be a clue to the presence of an omental cyst [8].

Treatment is complete excision. While there have been multiple reports of laparoscopic resection of omental/mesenteric cysts [1, 9, 10], open resection remains an appropriate course if there is diagnostic uncertainty, inability to safely perform laparoscopic resection, or an inability to completely excise the cyst without spillage of its contents. Previously published literature notes that omental cysts are less likely to be adherent to neighboring viscera and thus less likely to require bowel resection. However, in our case, the cyst was intimately associated with the tail of the pancreas. This increased the difficulty of the operation, but did not ultimately require pancreatectomy.

Our patient had a primary omental cyst. Like many patients who are ultimately found to have an omental or mesenteric cyst, the patient presented with abdominal pain and the initial clinical suspicion was for a more common abdominal pathology—in this case, the surgical team was consulted with concern for appendicitis—until subsequent imaging revealed the true diagnosis. In a previous series from our institution this was a typical sequence of events leading to the diagnosis of omental/mesenteric cyst [11]. The acuity of this patient’s symptoms is less common compared to the gradual onset of abdominal distention that typically heralds a developing omental cyst [12]. CT of the abdomen and pelvis was consistent with an omental cyst, given the cyst’s anterior location. Mesenteric cysts are characteristically surrounded by small bowel on imaging. Though immunohistochemistry was not performed to definitively characterize the nature of the cyst, gross pathology and operative findings suggest a complex mesothelial cyst. Though the rarity of these masses complicates efforts to characterize their behavior, the literature does note a propensity for mesothelial cysts to be found in the omentum, to be unilocular in nature, to originate from the transverse mesocolon, and to contain serous fluid [13, 14]. These criteria are in keeping with characteristics of our patient’s cyst.

## Conclusion

This 4-year-old child presented with abdominal pain, gradually increasing abdominal distention, and subclinical early satiety. He was found to have an omental cyst and underwent successful surgical resection. Though a benign mass with minimal chance of recurrence after complete excision, this cyst’s intimate relationship with the pancreas and the careful dissection required to separate those two structures highlights the occasionally challenging management of this rare finding.

## Data Availability

Not applicable.

## Abbreviation

WBC: White blood cells
